# Bedside Assessment of the Kidneys and Bladder Using Point of Care Ultrasound

**DOI:** 10.24908/pocus.v7iKidney.15347

**Published:** 2022-02-01

**Authors:** Patrick J Taus, Surya Manivannan, Ria Dancel

**Affiliations:** 1 Department of Internal Medicine, Division of Nephrology and Hypertension, University of North Carolina School of Medicine Chapel Hill, North Carolina; 2 Departments of Internal Medicine and Pediatrics, University of North Carolina School of Medicine Chapel Hill, North Carolina

**Keywords:** point of care ultrasound, nephrology, renal, kidney, bladder

## History and Background

Given the contrasting echogenic characteristics of the urinary system and their easily identifiable distortion in response to numerous pathologic processes, the sonographic examination of the kidney and bladder can provide a wealth of clinical information 1, 2. Although performed for decades as a referral and comprehensive radiologic study, improvement in the cost and performance of portable ultrasound devices has now made point of care ultrasound (POCUS) accessible to a growing number and variety of healthcare providers. The purpose of this review is to describe the technique and benefits of using POCUS to evaluate the kidneys, ureters, and bladder in common clinical scenarios.

POCUS gives clinicians more diagnostic information at the bedside than they can gather using traditional physical examination techniques, allowing them to expedite further diagnostic tests and provide more targeted and timely management. For example, while a distended bladder may be palpated on physical exam, the positive likelihood ratio for palpation of 600 mL of urine is only 1.62 when using POCUS as the criterion standard 3. Furthermore, POCUS can identify causes of bladder outlet obstruction, such as an obstructed or malpositioned urinary catheter or a bladder mass, thus informing appropriate management. While referral ultrasound is the standard against which POCUS is compared, it is not always immediately available due to resources or patient condition. POCUS can be performed at the bedside within minutes, does not subject patients to radiation, and can be easily repeated to gauge the progression of pathology or impact of interventions 4.

Ultrasound machines are becoming more portable and inexpensive, placing them in the hands, and pockets, of more clinicians. While pocket ultrasounds generally have inferior resolution compared to full platform machines, particularly in obese patients, a 2017 study comparing pocket POCUS performed by urology residents to standard sonographer-performed ultrasound for presence of hydronephrosis resulted in a Cohen’s kappa coefficient of 0.62, suggesting moderate agreement 4, 5. A similar and more recent study showed moderate to strong agreement (κ = 0.61-0.80) for measurement of kidney length and presence of cysts or hydronephrosis 4. These studies support the use of pocket devices to improve diagnosis at the bedside while cautioning against overconfidence in it as a standalone imaging modality.

## Technique and normal sonographic appearance of the kidney

Ultrasound evaluation of the urinary system consists of evaluation of the kidneys, bladder, and the ureters near the ureteropelvic (UPJ) and ureterovesical (UVJ) junctions, typically using a 2 - 5 MHz transducer in B-mode with the patient in a supine or decubitus position 1, 2. While a phased array transducer fits well between ribs, the large footprint of the curvilinear transducer allows for easier longitudinal measurement of the kidneys and bladder 2. Color Doppler techniques can be used to evaluate arterial inflow and venous drainage 6, 7, 8, 9. The right kidney can be evaluated through an acoustic window provided by the liver, typically anteriorly or along the mid-axillary line. The left kidney is approximately 1 vertebral level higher than the right, can be visualized through an acoustic window provided by the spleen, and typically requires a more posterior approach 10. The marker on the transducer should initially be oriented superiorly and then slowly rotated 15 - 30 degrees (counterclockwise for the right kidney and clockwise for the left kidney) to obtain the true longitudinal plane of the obliquely positioned kidneys, since the inferior poles are more anterolateral 2. Measurement of kidney length should be obtained multiple times and the longest measurement recorded. Rotation of the transducer 90 degrees counterclockwise from the longitudinal plane yields the short axis view. Fanning from the superior pole to inferior pole in the transverse plane ensures no pathology is missed. To aid in visualization, a seated position can help move the left kidney below the costal margin as can a prolonged inspiratory breath-hold 10, 11.

**Figure 1:**
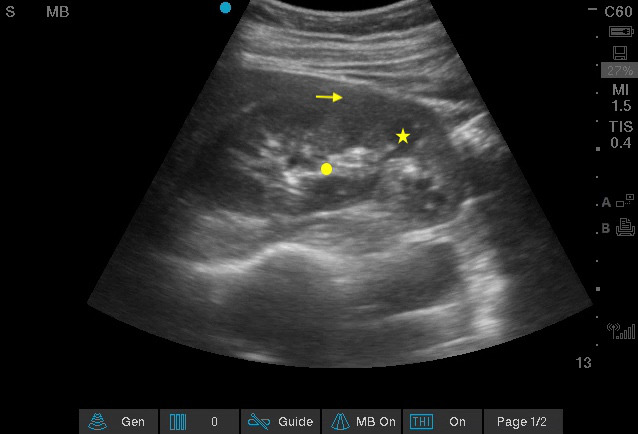
Normal right kidney in longitudinal view. Arrow: cortex, star: hypoechoic medullary pyramid, dot: renal sinus with hyperechoic fat. Video S1 can be found in supplementary material.

The normal kidney has a characteristic ultrasonographic appearance (Figure 1, Video S1). Typically, a layer of white perinephric fat surrounds the darker cortex of the kidney. The cortex appears more echogenic than the medulla, while appearing hypo- or isoechoic compared to the liver and hypoechoic to the spleen 10, 12, 13. The increased echogenicity of a cirrhotic liver alters this relationship, decreasing the sensitivity of detecting abnormal right kidney pathology. The tips of the pyramids, oriented toward the renal sinus, form the papilla that excrete urine into minor calyces, which fuse to form major calyces and then subsequently the renal pelvis. In younger patients the pyramids in the medulla can be mistaken for renal cysts due to their relative hypoechogenicity 14. The renal sinus contains the collecting system, the large renal vessels, and perihilar fat. When urine is draining normally, the renal sinus appears hyperechoic on ultrasound owing to the predominant echogenicity of fat 11, 14, 15.

Emamian et al. showed in a study of 665 healthy volunteers that the measurement of renal length alone is more practical than the calculation of renal volume from ultrasound measurements of kidney length, width, and thickness and provided reference values from their large data set 16. The volume and length of the right kidney is typically smaller than the left. The authors grouped volunteers and their kidney measurements by chromosomal sex. In adults, the median length of the right kidney in females is 10.7 cm (range 9.5 - 12 cm) and in males, 11.2 cm (range 10.1 - 12.4 cm). The median length of the left kidney in females is 11 cm (range 9.4 - 12 cm) and median length in males is 11.5 cm (range 10.4 - 12.6 cm). The authors noted a small but acceptable difference between supine and prone measurements 16.

## Ultrasonographic appearance of chronic kidney disease

**Figure 2:**
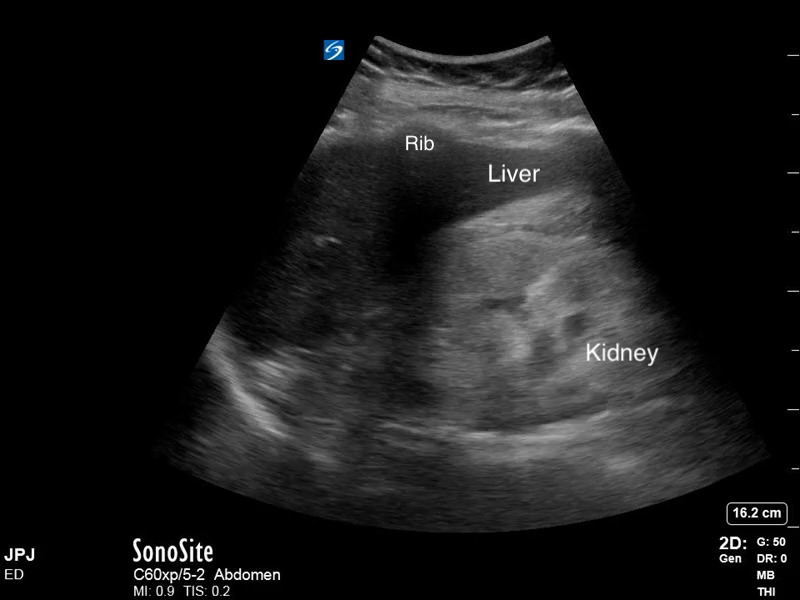
Hyperechoic kidney seen in a patient with chronic kidney disease. Note that the kidney echogenicity is described relative to the liver parenchyma. Video S2 can be found in supplementary material.

Renal length, cortical echogenicity, and cortical thickness can give clues to the chronicity of medical renal disease. Keeping in mind that people of shorter stature tend toward the lower range of kidney length, kidneys smaller than the lower end of the ranges described above suggest chronic kidney disease (CKD) 16. Certainly, a length of < 9 cm is suspicious 17. As mentioned previously, the transducer needs to be rotated slightly obliquely to capture the longest orientation of the kidney as failure to do so will lead to an underestimation of kidney length. Regardless of the cause of the pathology, the cortex becomes more echogenic with respect to the appearance of the nearby liver or spleen with prolonged pathology^11^ (Figure 2 and Video S2). Ideally, the thickness of the cortex should be measured from the base of the pyramids, if visible, to the capsule. If the pyramids are not easily visualized, the parenchyma can be measured from the hyperechoic renal sinus to the capsule 11. Normal cortical thickness is 7 - 10 mm and normal parenchymal thickness is 15 - 20 mm 18. In general, short kidney length with cortical thinning is suggestive of advance CKD; however, these sonographic signs should be considered in totality and in context of historical clues as hyperechogenicity can be seen in the setting of acute tubular necrosis. Additionally, POCUS can readily uncover underlying cystic disease associated with CKD (as discussed further below).

## Ultrasonographic appearance of obstructive kidney disease

**Figure 3:**
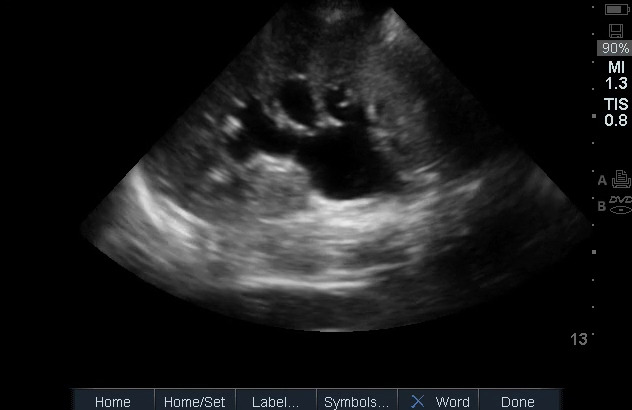
Moderate hydronephrosis with the classic bear claw appearance caused by dilation of the major and minor calyces. Video S3 can be found in supplementary material.

**Figure 4:**
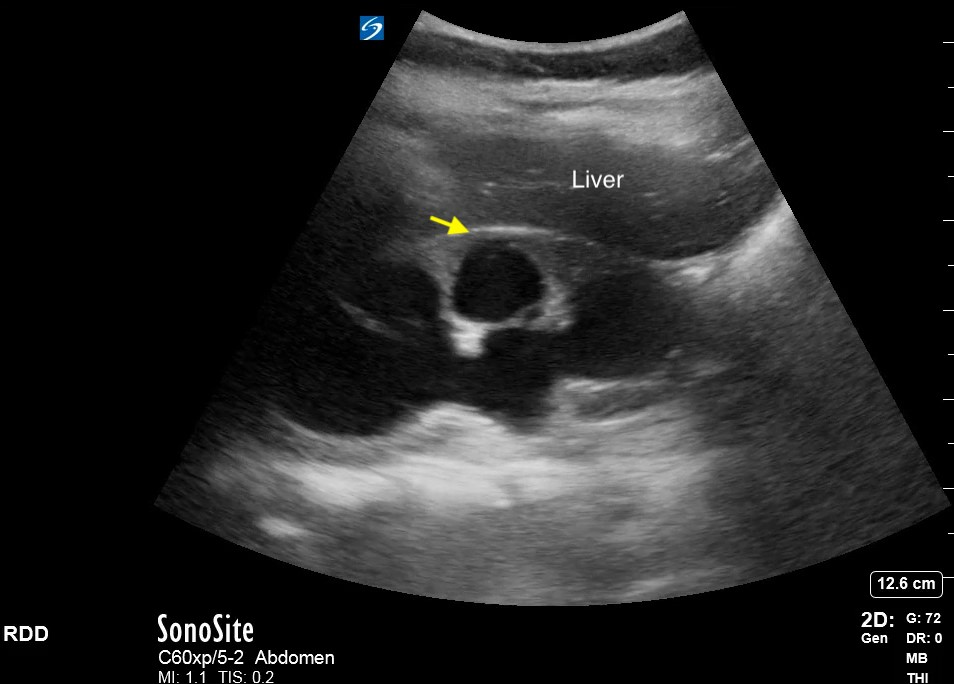
Severe hydronephrosis with ballooning of the calyces and effacement of the renal cortex (yellow arrow). Video S4 can be found in supplementary material.

Acute kidney injury (AKI) due to obstruction can be suggested by the finding of hydronephrosis on POCUS. Typically, the collecting system and ureters are actively drained of urine, such that the predominant signal in the renal hilum is hyperechoic due to sinus fat. In the setting of obstructive uropathy, distention of the collecting system with anechoic urine (i.e., hydronephrosis) can be readily detected by ultrasound. Hydronephrosis is graded as mild when only the renal pelvis and/or major calyces are dilated, moderate when both major and minor calyces appear dilated (the bear claw appearance seen in Figure 3 and Video S3), and severe when there is parenchymal effacement of the cortex^15^ (Figure 4 and Video S4). Hydronephrosis can be distinguished from hypoechoic pyramids by the presence of interdigitating hyperechoic sinus fat between the dilated calyces, and distinguished from renal vasculature with the use of color Doppler, which should show no flow in the collecting system 15. Mild hydronephrosis may be seen in non-obstructed states such as pregnancy, full bladder, vesico-ureteral reflux, urinary tract infection, and brisk diuresis 19.

Numerous studies have evaluated the use of POCUS for detection of hydronephrosis with sensitivities and specificities ranging from 72 - 96% and 59 - 96%, respectively 20, 21, 22, 23, 24, 25, 26, 27. A great majority of studies to date have focused on patient populations presenting with flank pain or suspicion for renal colic with the bedside ultrasounds performed by emergency physicians 20, 21, 24, 25, 26, 27, 28. Nixon et al. studied POCUS performed by 28 generalists in rural New Zealand hospitals as part of routine practice and found a high sensitivity (90%) and specificity (96%) for hydronephrosis, which suggests POCUS evaluation for hydronephrosis may be feasible in other settings 22. Ensuring adequate intravascular volume with appropriate IV fluids augments the detection of hydronephrosis by POCUS by promoting urine production and subsequent dilation of the collecting system 29. 

## Technique for bladder imaging and measurement

**Figure 5:**
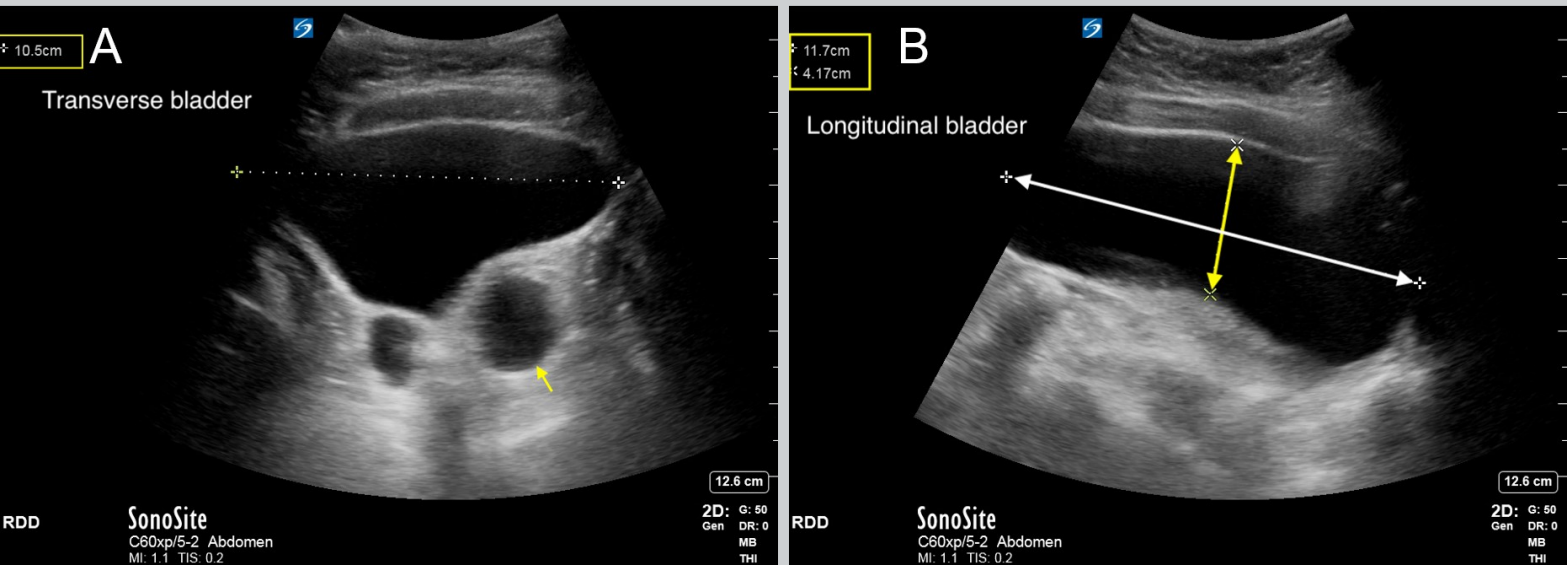
A) The width of the bladder is measured in the transverse view. In this image, the width is approximately 10.5 cm. Also seen in this image is a dilated left ureter as it enters the bladder at the UVJ (yellow arrow). Video S5a can be found in supplementary material. B) The depth (double ended yellow arrow) and height (double ended white arrow) of the bladder is measured in the longitudinal view. In this image, the depth is approximately 4.2 cm and the height is 11.7 cm. Using the width measurement in the above figure, this patient’s estimated bladder volume is 10.5 cm x 4.2 cm x 11.7 cm x 0.52 = 268 mL. Video S5b can be found in supplementary material.

The presence of hydronephrosis should prompt careful evaluation of the bladder. The bladder is imaged by placing the transducer in the anterior midline, directly above the pubic symphysis. With the transducer in a transverse plane, fanning superiorly to inferiorly allows for measurement of maximal width (Figure 5a and Video S5a). The transducer is then rotated 90 degrees to obtain a longitudinal view and provide for measurement of the bladder’s anterior-posterior depth and cranio-caudal height^30^ (Figure 5b and Video S5b). Several formulae allow for estimation of bladder volume based on these measurements with similar degrees of accuracy, the most common being the prolate ellipsoid formula:

Volume (mL) = depth x width x length x 0.52 31.

While imaging the bladder, the presence of ureteral jets can be captured by using the color or power Doppler mode with a small window and low color velocity while over the UVJ which appear as papules in the posterior bladder wall 14, 15. In addition, the UVJ should be evaluated for the presence of stones. While evaluating the bladder, avoid excessive far field gain as posterior acoustic enhancement can easily obscure findings posterior to the bladder such as hydroureter, ureteral stones, and shadowing from stones or free fluid 10. 

## Advanced POCUS renal evaluation for AKI

In patients presenting with kidney failure of unknown chronicity, a combination of small kidney length, increased echogenicity, and decreased cortical thickness can suggest CKD. It is important to note, however, that hyperechogenicity of the parenchyma can be seen in both AKI, where it may be due to inflammation or proteinaceous casts, as well as in CKD, where increased signal may be due to fibrosis 17, 19. Therefore, while hyperechoic renal parenchyma signals disease with 96% specificity and 67% positive predictive value, it gives no specific indication regarding the chronicity, severity, or etiology of intrinsic renal disease 19. Furthermore, since glomeruli only make up 8% of the cortex, even biopsy proven severe glomerular disease may not cause ultrasonographic changes 17.

More advanced POCUS techniques for investigation of AKI include interrogation of renal perfusion and drainage using Doppler-based modalities to calculate the renal resistive index (RRI) and venous impedance index (VII), respectively 7, 8, 32, 33, 34, 35. Using pulsed-wave Doppler interrogation of the interlobar arteries, the RRI is calculated using the formula:

RRI = (peak systolic velocity - end diastolic velocity) / (peak systolic velocity)

A greater difference between peak systolic velocity and end diastolic velocity leads to a higher RRI and reflects resistance to blood flow 19. Normal RRI ranges between 0.56 and 0.66 and was found to be > 0.7 in only 3% of healthy kidneys 36. While an advanced POCUS skill, two studies have shown that novice and intermediate POCUS users can obtain RRI measurements with good to excellent interobserver reproducibility compared to expert sonographers with only 4 - 6 hours of training 32, 34. Similarly, venous drainage from the kidneys can be assessed using pulsed-wave Doppler in the interlobar veins. If the intra-renal venous flow pattern is biphasic, VII can be calculated using the same formula as above 35. For both RRI and VII, averages should be calculated from multiple measurements. 

The clinical utility of RRI, intra-renal venous flow pattern, and VII remain unclear at this time. A 2015 meta-analysis of 449 patients with AKI suggested that RRI > 0.7 predicts persistence of AKI beyond 72 hours, although the authors noted significant heterogeneity of the studies and recommended further large prospective studies 6. Subsequently, Oliveira et al. prospectively found that RRI between critically ill patients without AKI and those with transient AKI were not statistically different but patients with persistent AKI had statistically higher RRI compared to the other two groups 37. Conversely, Darmon et al. showed that while patients with AKI had increased RRI compared to those who did not, RRI was poorly predictive of persistent AKI 7. Wiersema et al. found that there was no significant difference in measurements of both arterial and venous flow in critically ill patients with or without AKI 35. Similarly, Spiegel et al. found that abnormalities in interlobar venous flow patterns were not associated with increased risk of adverse kidney events 8. Further study of systemic congestion assessment is underway that incorporates renal perfusion and drainage with inferior vena cava (IVC) investigation and Doppler interrogation of the portal and hepatic veins 9.

## POCUS in the evaluation of renal colic

Renal colic is the term used to describe the abrupt and severe flank and low back pain caused by acute obstruction and distension of the ureter and renal pelvis 29. POCUS is a useful aide in the management of renal colic, allowing for the direct visualization of stones in some cases or providing indirect evidence of their presence by detecting signs of urinary tract obstruction such as hydronephrosis or absence of ureteral jets 38. 

**Figure 6:**
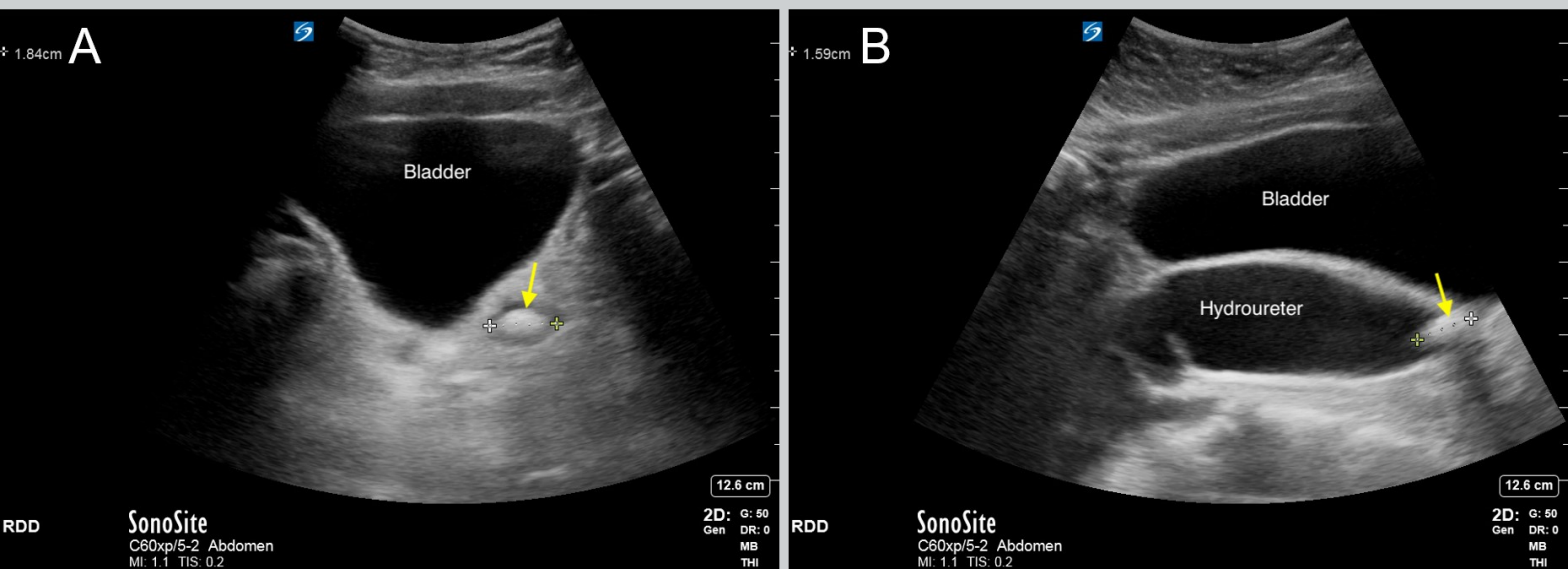
A) 1.8 cm stone in the UVJ (yellow arrow), seen in transverse. Video S5a can be found in supplementary material. B) Stone in the UVJ (yellow arrow), seen in longitudinal and measuring approximately 1.6 cm. Note that the ureter is severely dilated. Video S5b can be found in supplementary material.

Direct visualization of calculi within much of the course of the ureter can be difficult as overlying bowel gas and adjacent bony structures leads to poor insonation. As a result, only stones near the UPJ or UVJ are amenable to sonography, leading to only about 64% of stones being in the field of view afforded by POCUS^39^ (Figures 6a and 6b and Videos S5a and S5b). Furthermore, ultrasound has a sensitivity of 16% for stones smaller than 7 mm and 75% for stones > 7 mm 40. This limited field of view coupled with the poor sensitivity of ultrasound for small stones makes POCUS of limited value for detecting renal calculi (sensitivity of 24% for all stones compared to computed tomography) 40. On the other hand, the high specificity (98%) and positive predictive value suggests that calculi identified on ultrasound correspond to calculi visualized on computed tomography (CT) and therefore is an appropriate technique to rule-in the presence of stones 40. Even without direct visualization of stones, when initial clinical suspicion is high and a strong competing diagnosis is lacking, the presence of hydronephrosis on POCUS is supportive of renal colic.

Despite the limitation in direct stone visualization, the presence and grade of hydronephrosis can help to inform the management of patients presenting with renal colic. Patients with no hydronephrosis are at low risk for hospitalization or urologic intervention 41. Moderate to severe hydronephrosis has poor sensitivity but 94% specificity for the presence of a stone 38. Patients with renal colic and moderate or severe hydronephrosis on POCUS are more likely to require a urologic intervention 38. The improved sensitivity of diagnosing larger stones when a high degree of hydronephrosis is present may be helpful for stratifying a patient’s risk for failure of expectant management of urolithiasis and support a clinician’s choice to pursue further imaging or urologic consultation if symptoms do not resolve after a trial of passage 38. Limitations of using hydronephrosis in the diagnosis of renal colic is that a proportion of renal stones do not cause hydronephrosis, and the presence of hydronephrosis does not rule out alternative diagnosis for abdominal pain 38. Of note, if bilateral hydronephrosis is seen in the investigation of renal colic, other etiologies, associated with a lower urinary tract obstruction, should be considered although simultaneous ureteral obstruction is possible 42. Patients with renal colic can be severely volume depleted due to associated nausea and vomiting, leading to a false-negative exam for hydronephrosis. Sensitivity for identifying hydronephrosis can be increased by appropriate volume resuscitation prior to imaging 29.

Color Doppler ultrasonography (CDU) at ureterovesical junctions can be used to look for ureteral jets of urine flowing into the bladder which serve as a surrogate for ureteral flow. In combination with the presence of hydronephrosis, ureteral jet asymmetry with an absent or weaker jet lateralizing to the side with symptoms of renal colic can be seen in obstructive ureterolithiasis. Notably, bedside ureteral jet evaluation does not significantly contribute to prediction of patient outcomes, including need for hospitalization or urologic procedure. It can, however, be used by clinicians to assess the need for additional advanced imaging 43. 

CDU twinkling artifact is a form of intrinsic noise when insonating certain rough reflective surfaces (like urolithiasis), and appears as a discrete focus of alternating color signals behind an echogenic surface 44. The presence of a twinkle may indicate the presence of nephrolithiasis. In a recent meta-analysis, the pooled sensitivity of this sign was 88% with a range of 40-100% and the pooled specificity was 79% with the same range, suggesting significant heterogeneity 44. 

According to a 2018 meta-analysis, accuracy of POCUS for the diagnosis of nephrolithiasis is modest relative to CT scan, with a pooled sensitivity and specificity of 70.2% and 75.4%, respectively 38. The decision to pursue further imaging after POCUS should be informed by a combination of history, physical exam, and urine analysis, in addition to POCUS findings. Encouragingly, studies comparing ultrasound versus CT in the initial management of renal colic show no difference in complications, adverse events, pain scores, or hospitalizations 45. 

## Utility of POCUS in urinary tract infection:

Although pyelonephritis can appear as areas of increased or decreased echogenicity relative to the surrounding kidney and Doppler evaluation can demonstrate decreased vascularity due to corresponding tubular ischemia, the sensitivity of US for detection of pyelonephritis is low 46.The main utility for POCUS in the evaluation of patients with pyelonephritis is the timely identification of underlying obstructive uropathy and complications such as pyonephrosis, abscess, or emphysematous pyelonephritis, which can expedite appropriate treatment. Chen et al. found retrospectively that POCUS detected significant abnormalities such as stones, hydronephrosis, or abscesses in 39.6% of patients presenting with acute pyelonephritis 47. The detection of significant findings increased to 56.6% of patients with the addition of abdominal radiography which was nearly equal to the 58.8% of patients found to have significant abnormalities by CT 47. Use of POCUS in this study diverted 34.3% of patients to receive interventions such as abscess drainage, stone removal, nephrostomy, and nephrectomy 47.

Evidence of emphysematous pyelonephritis typically appears as a high amplitude/echogenic focus associated with distal “dirty shadowing” or low-level echoes and reverberations caused by the near total sound reflection at the tissue-air interface, distinct from the echo-free shadow formed by calculi 48, 49. The ultrasonographic appearance of renal abscesses can range from hyper- to hypoechoic focal masses or cystic structures, potentially with posterior acoustic enhancement 46. The absence of vascularity helps to distinguish abscesses from potential malignant findings, although occasionally mobile debris may be seen that can be misinterpreted as vascular flow on Doppler 46, 50. If air is present within the fluid collection, a ring-down artifact may be present 51. It should be noted that while POCUS can suggest emphysematous changes or abscess, the most recent recommendation of the American College of Radiology maintains CT as the imaging study of choice for patients presenting with pyelonephritis and either a complex presentation or failure to respond to antibiotics 52.

## Mimics, masses, and variants 

Renal cysts are common and can be found in 24 - 47% of the general population, with a higher prevalence in the aging population 53. Cysts appear as circular or ovoid shaped, typically anechoic structures that demonstrate posterior acoustic enhancement 10, 15. Although commonly located in the periphery of the kidney, cyst can be found in the sinus where they can be mistaken for hydronephrosis. Because ultrasound can accurately detect cysts larger than 0.5 cm, POCUS practitioners will encounter cysts and must be able to characterize and distinguish them from similar lesions, such as hydronephrosis, solid masses, and abscesses 54. While hydronephrosis progresses from the renal pelvis to the major and minor calyces and then to the cortex, cysts are cortical or parapelvic and do not follow this anatomic pattern 55, 56. Most cysts are asymptomatic. However, ruptured cysts can cause pain and hematuria and cortical cysts may grow large enough to stretch the renal capsule 56. Obstruction is another possible, though rarer, complication 56.

**Figure 7:**
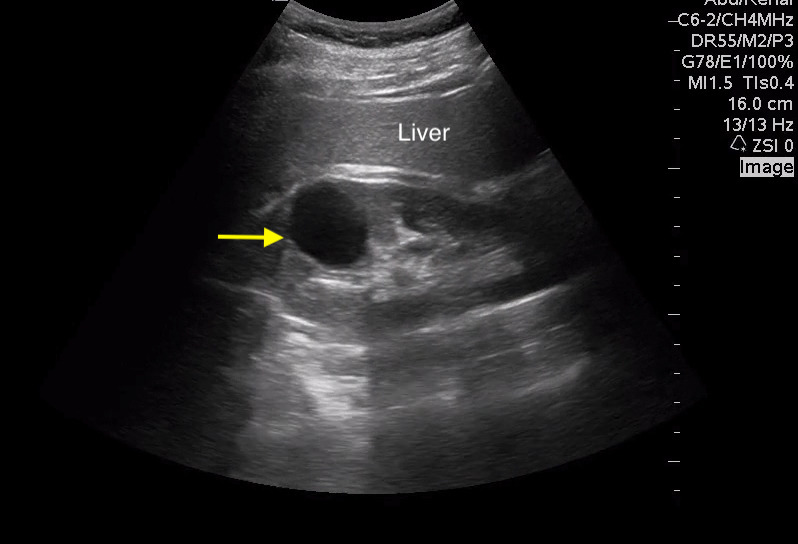
Simple renal cyst in right kidney (yellow arrow). Note the posterior acoustic enhancement. Video S6 can be found in supplementary material.

While it should be noted that the Bosniak classification of renal cysts was created to apply to lesions seen on renal CT, the 2019 version of the Bosniak classification does allow diagnosis of simple cysts—Bosniak 1—by ultrasound if the structure is round or oval, thin-walled, anechoic, not septated, and displays posterior acoustic enhancement 57. The posterior acoustic enhancement provides reassurance that the cyst contains simple fluid (Figure 7 and Video S6). These simple cysts need no further evaluation 1. All other cystic lesions should be referred for more definitive imaging 57.

**Figure 8:**
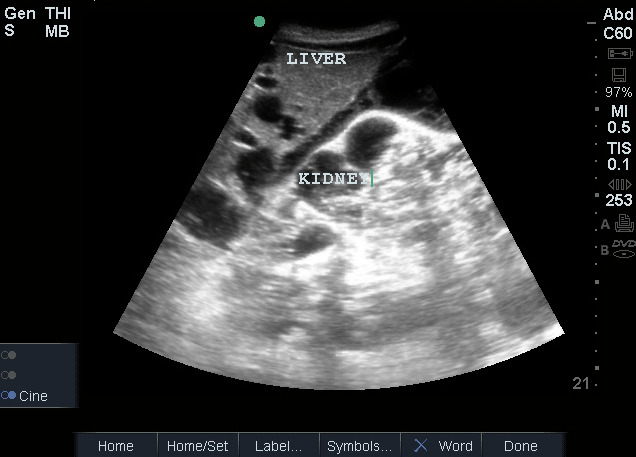
Autosomal dominant polycystic kidney disease causing numerous cysts in the right kidney and liver.

The most common cystic kidney diseases are autosomal dominant polycystic kidney disease (ADPKD) and acquired cystic kidney disease (ACKD). Both diseases are characterized by multiple bilateral kidney cysts. However, while ADPKD can progress to end stage kidney disease, ACKD increases in prevalence the longer patients have advanced CKD or end stage kidney disease–reaching a prevalence of almost 90% in patients who have been on dialysis for 5 - 10 years 58, 59. POCUS can easily detect two features that distinguish ADPKD and ACKD–renal size and extra-renal involvement. ADPKD invariably results in enlarged kidneys while kidneys with acquired cystic disease are frequently smaller, with cortical thinning typical of CKD 60. In patients with ADPKD, Bhutani et al. showed that kidney length measured by ultrasound had an area under the receiver operating curve (AUROC) similar to magnetic resonance imaging (0.87 versus 0.86, respectively) for predicting CKD stage 3 within 8 years, using a cutoff of 16.8 cm on ultrasound 61. Finally, given their proximity to the kidneys, extra-renal manifestations of ADPKD in the liver and spleen are easy to assess with POCUS. Liver cysts are especially common–affecting over 80% of patients with ADPKD at 30 years old^62^ (Figure 8). Distinguishing ADPKD from ACKD is important as ACKD is a pre-malignant condition 1.

Solid renal masses may have cystic components but are mostly echogenic. The most common tumor is renal cell carcinoma, which is typically found in the periphery and may be somewhat difficult to identify since they are usually isoechoic and are poorly differentiated from the surrounding parenchyma 11. Other malignant tumors include transitional and squamous cell carcinomas, adenocarcinomas, and lymphoma 11. The most common benign mass is angiomyolipoma (AML), which is distinguishable from RCC if fat is a major component as it will be hyperechoic. However, it is important to remember that AML may also have minimal fat and therefore appear similar to RCC on POCUS 63. Overall, the sensitivity of ultrasound is inadequate for detection of RCC and CT imaging or alternatively MRI remain the preferred imaging modalities. 

**Figure 9:**
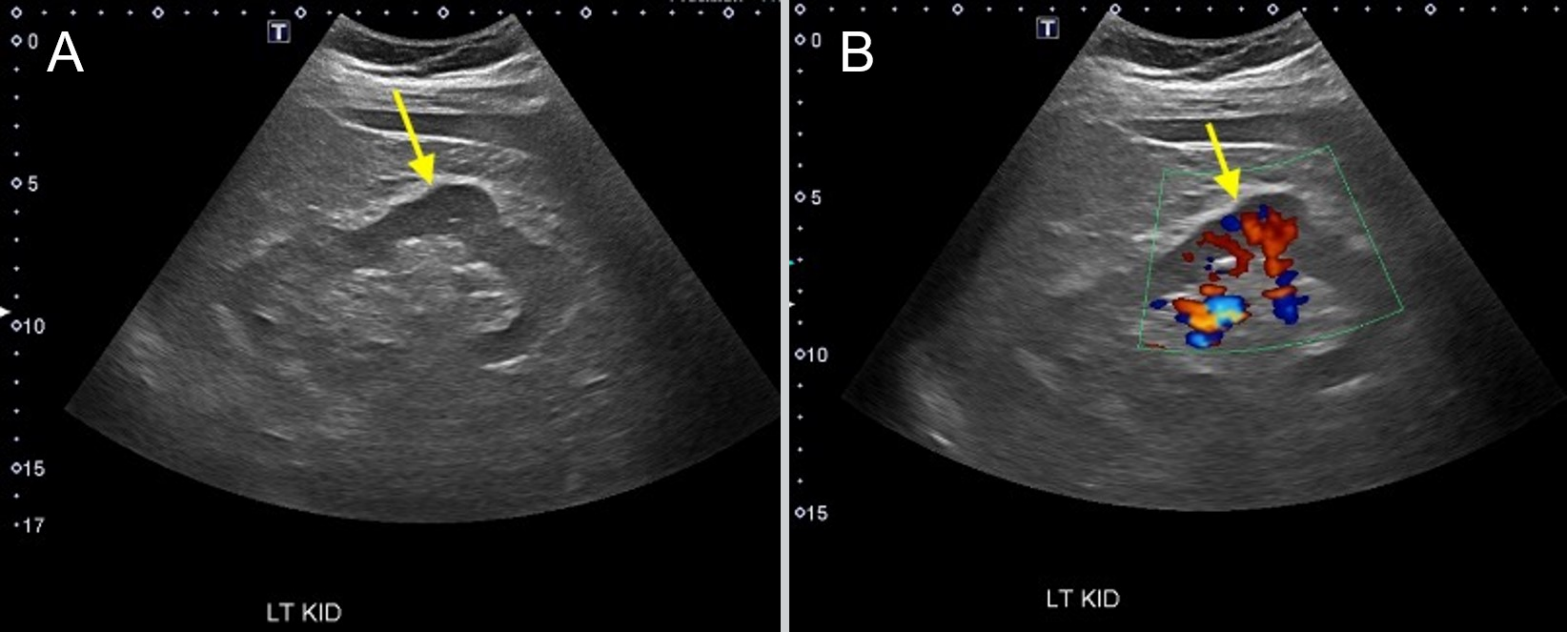
A) Dromedary hump (yellow arrow) of the left kidney. Image courtesy of Dr. AbhilashKoratala. B) Dromedary hump (yellow arrow) of the left kidney. Color flow Doppler illustrates normal blood flow into this normal cortical parenchyma. Image courtesy of Dr. Abhilash Koratala.

**Figure 10:**
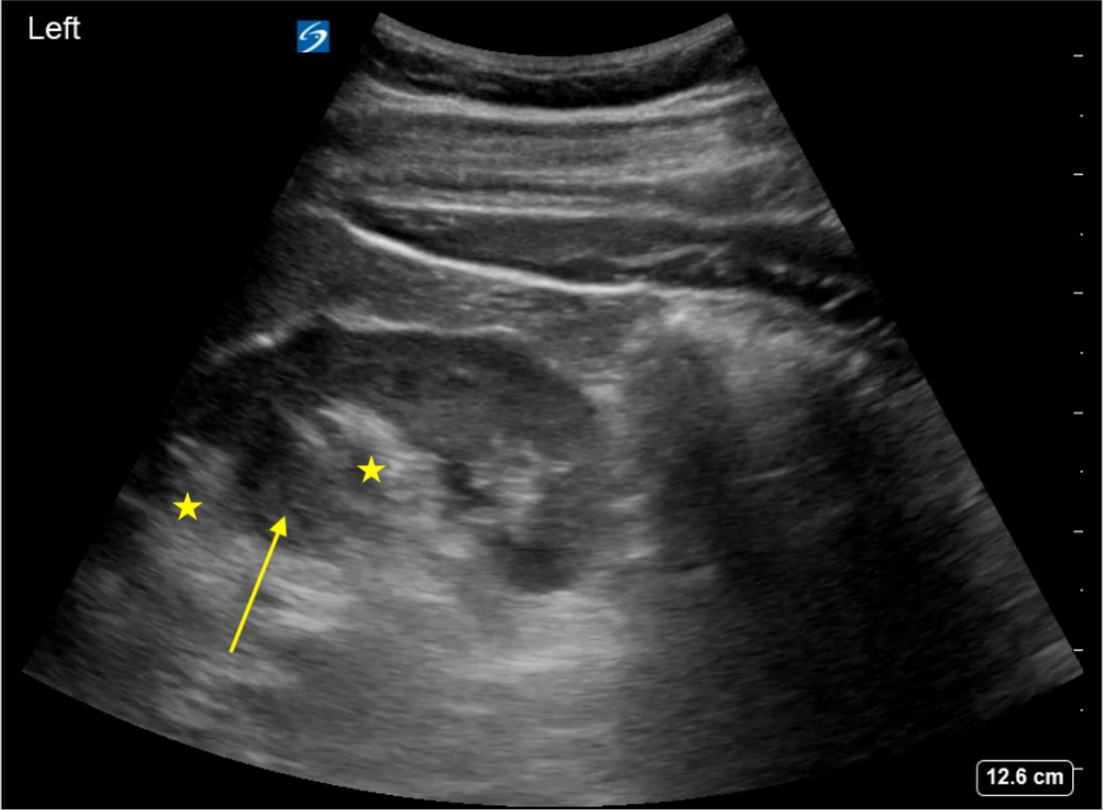
Hypertrophied column ofBertin (yellow arrow). Note that the hypertrophied cortical parenchyma appears to cleave the fat in the renal pelvis (yellow stars). Image courtesy of Dr. Kylie Baker.

**Figure 11:**
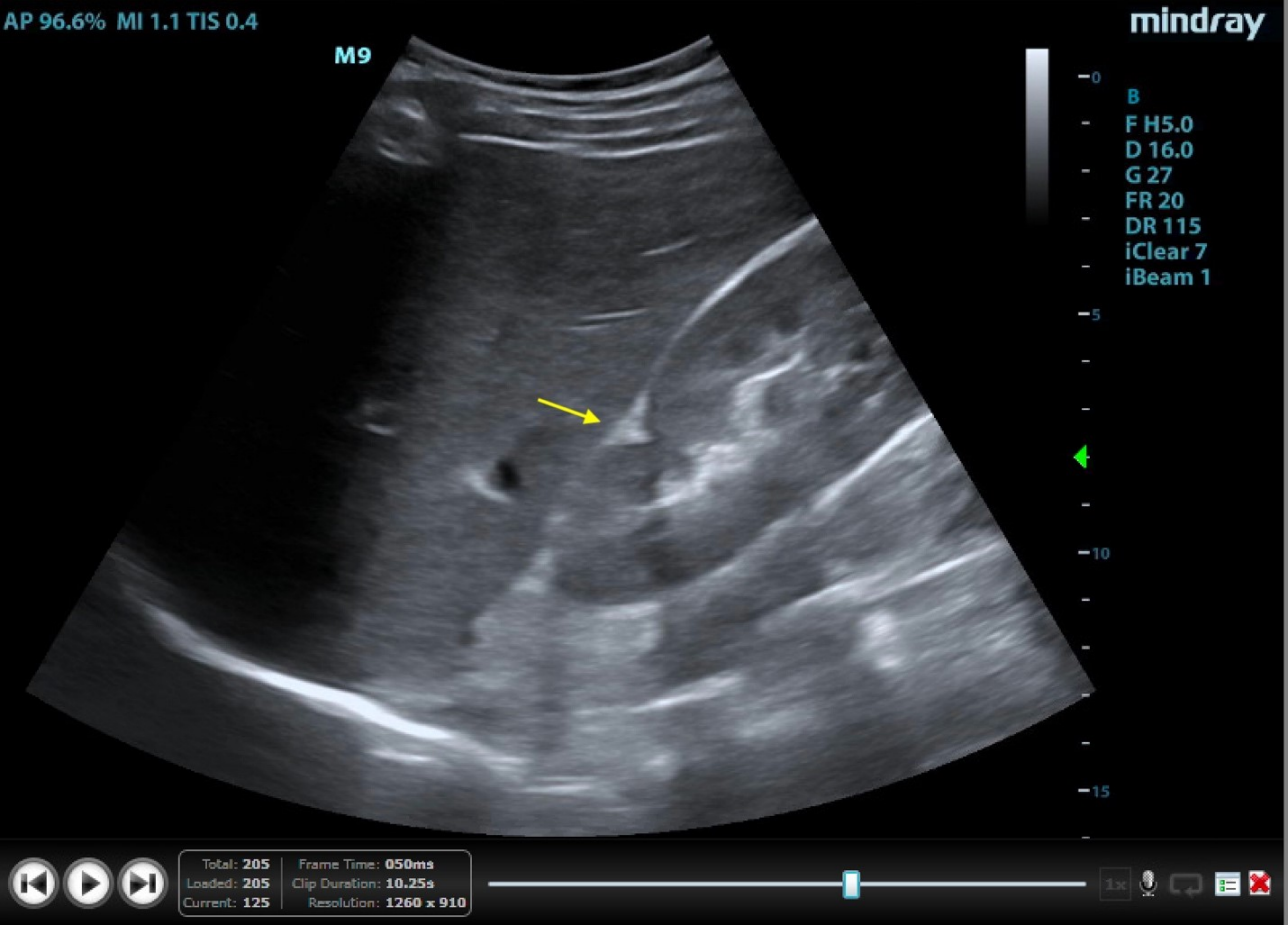
Junctional parenchymal defect (yellow arrow). Video S7 can be found in supplementary material.

Other common ultrasonographic findings easily mistaken for neoplasms may be developmental variants, infections, or vascular abnormalities 64. The dromedary hump is one such common normal variant seen on POCUS (Figure 9a and 9b). It is a protuberance on the lateral aspect of the left kidney that resembles a camel’s hump that is thought to be a result of being molded by the spleen 65, 66. Hypertrophied column of Bertin is another common variant that is characterized by a prominent, wide bridge of cortical parenchyma between pyramids that appears to bulge into the renal pelvis and mimics a mass^64^ (Figure 10). Both variants can be recognized by their position in the kidney and their similarity in appearance with the rest of the normal cortical parenchyma. Junctional parenchymal defects occur due to partial fusion of fetal renunculi and may be mistaken for a hyperechoic lesion or scar^67^ (Figure 11 and Video S7). Finally, persistent fetal lobulations are caused by indentations that characteristically overlie the spaces between the pyramids and may lead to an appearance of multiple mass-like projections in the cortex 64. Previously discussed infectious processes, such as focal pyelonephritis and abscesses can also mimic neoplastic processes. Finally, vascular defects, the most common being arteriovenous malformations, may mimic tumors as well. These may appear anywhere in the kidney and will look similar to malignant tumor on CT as there will be contrast enhancement. However, color Doppler enhanced POCUS will show that this “tumor” is a collection of blood vessels without parenchyma 64. Despite the ability of POCUS to differentiate some of these neoplastic mimics, any beyond a clear simple cyst or common anatomical variant requires referral. 

As more POCUS exams are performed, more incidental renal findings will be found. Responsible practice of POCUS involves recognizing and reporting these findings. A study of 1,452 focused assessment with sonography for trauma (FAST) exams in 2 academic emergency departments showed that incidentalomas were found in 9.4% of patients and the most common incidental findings were renal cysts 68. Of those, only 22.6% were documented and only 6 of 70 discharged patients had documentation that they were notified of the incidental finding or were referred for follow-up 68. A small case series of incidental renal cell carcinomas seen in an ambulatory cardiology clinic suggests that widespread adoption of POCUS coupled with robust referral may result in early detection and improved outcomes 69.

## POCUS Training

POCUS is rapidly diffusing throughout general internal medicine and its subspecialities throughout the training spectrum from undergraduate and graduate medical education to subspecialty fellowship training and continuing medical education 70. Over half of medical schools surveyed in 2014 included POCUS in their curriculums and that number has undoubtedly increased since then 71. The Alliance of Academic Internal Medicine (AAIM) released a position statement in 2019 describing the barriers to creating a POCUS curriculum and offering a model for longitudinal training 70. That same year, POCUS became a “mandatory skill” for residents in the Netherlands 72. A national survey showed that these residents thought renal ultrasound was the second most useful core application, after IVC ultrasound. The most important barrier they identified was an insufficient number of experts to teach the curriculum 72.

Nephrologists who are expert at not just renal and bladder ultrasound, but who can also integrate focused cardiac, lung, and vascular ultrasound are in a good position to teach all levels of learners. Nephrology fellowships are beginning to integrate POCUS into training, and a model curriculum has been proposed 65. Introductory courses and workshops are increasingly being offered at society conferences 1. Those who wish to pursue competency in renal and bladder sonography may pursue certification through the American Society of Diagnostic and Interventional Nephrology. For more comprehensive training, the Society of Hospital Medicine and the American College of Chest Physicians offer a certificate of completion in acute and critical care POCUS. 

## Conclusions and Future Direction

The use of POCUS for the assessment of urinary system pathology is poised to greatly expand in the coming years given the declining cost and improving performance of the ultrasound equipment itself, the proliferation of POCUS training at all levels of medical education, the significant savings it affords compared to traditional types of imaging, and most importantly the additional clinical information it provides at the bedside. It is unknown at this time if the previous sensitivities and specificities found in studies of traditional or emergency provider performed ultrasonography will be recapitulated by members of other specialties; however, one would expect that the ultimate performance characteristics will depend largely on the operator’s knowledge and experience, therefore, the early introduction of urinary system focused POCUS training along with incentivization for its use will likely lead to better performance and, in turn, improved patient care. For now, the utility of POCUS in the workup of kidney injury to quickly identify obstruction in the form of hydronephrosis and/or bladder distention and evidence of chronic renal disease is clear. Although POCUS may not supplant more advanced imaging for some indications given the factors mentioned above, the frequency with which it is able to preempt patients from requiring those forms of imaging will continue to grow in the coming years.

## Disclosures

There are no financial conflicts of interest for any of the contributing authors.

## Supplementary Material

 Video S1 Normal right kidney in longitudinal view (Figure 1). The transducer is being fanned ventrally to dorsally.

Video S2 Hyperechoic kidney seen in a patient with chronic kidney disease. Note that the kidney echogenicity is described relative to the liver parenchyma (Figure 2).

Video S3 Moderate hydronephrosis with the classic bear claw appearance caused by dilation of the major and minor calyces (Figure 3).

Video S4 Severe hydronephrosis with ballooning of the calyces and effacement of the renal cortex (Figure 4). The transducer is being fanned ventrally to dorsally.

Video S5a Transverse view of the bladder, fanning superiorly to inferiorly. Fanning allows identification and measurement of the widest diameter (Figure 5a). This video also shows a large stone in the left UVJ with associated hydroureter (Figure 6a).

Video S5b Longitudinal view of the bladder, fanning from patient’s right to left. Fanning allows identification and measurement of the longest anterior-posterior depth and cranio-caudal height (Figure 5b). This video also shows the large UVJ stone seen previously in a different plane (Figure 6b).

Video S6 Longitudinal view of right kidney with a simple cyst in the superior pole. Note the posterior acoustic enhancement (Figure 7).

Video S7 Longitudinal view of the right kidney with a hyperechoic cleft-like junctional parenchymal defect in the superior pole (Figure 11). The transducer is being fanned ventrally to dorsally.
